# Using fMRI to Assess Brain Activity in People With Down Syndrome: A Systematic Review

**DOI:** 10.3389/fnhum.2020.00147

**Published:** 2020-04-27

**Authors:** Maria Carbó-Carreté, Cristina Cañete-Massé, Maribel Peró-Cebollero, Joan Guàrdia-Olmos

**Affiliations:** ^1^Department of Cognition, Development and Educational Psychology, Faculty of Psychology, University of Barcelona, Barcelona, Spain; ^2^Institute of Neuroscience, University of Barcelona, Barcelona, Spain; ^3^Department of Social Psychology and Quantitative Psychology, Faculty of Psychology, University of Barcelona, Barcelona, Spain; ^4^UB Institute of Complex Systems, University of Barcelona, Barcelona, Spain; ^5^Quantitative Psychology Research Group (SGR 266), Generalitat de Catalunya, Barcelona, Spain

**Keywords:** down syndrome, fMRI, brain signal, brain activity, systematic review

## Abstract

**Background:** In the last few years, many investigations have focused on brain activity in general and in populations with different pathologies using non-invasive techniques such as electroencefalography (EEG), positron emission tomography (PET), functional magnetic resonance imaging (fMRI) and magnetic resonance imaging (MRI). However, the use of non-invasive techniques to detect brain signals to evaluate the cognitive activity of people with Down syndrome (DS) has not been sufficiently addressed. The objective of this study is to describe the state-of-the-art in fMRI techniques for recording brain signals in people with DS.

**Method:** A systematic review was performed based on PRISMA recommendations; only nine papers on this topic have been published. Three independent researchers selected all relevant information from each paper. Analyses of information concordance showed a high value of agreement between researchers.

**Results:** Although few relevant works have been published, the use of fMRI in people with DS is becoming an appropriate option to study brain function in this population. Of the nine identified papers, five used task designs, and four used resting-state paradigms.

**Conclusion:** Thus, we emphasize the need to incorporate rigorous cognitive activity procedures in evaluations of the DS population. We suggest several factors (such as head correction movements and paired sample techniques) that must be considered when designing an fMRI study with a task or a resting-state paradigm in a DS population.

## Introduction

Analysis of the cognitive activity of people with Down Syndrome (DS) is extraordinarily relevant, and it has become a foundation for better understanding the development of neurodegenerative diseases, mainly Alzheimer's disease (AD) (Prasher et al., 1996; Lamar et al., 2011; Neale et al., 2018; Pujol et al., 2018; Musaeus et al., 2019); studying the development and morphological characteristics of the brains of individuals with DS (Baburamani et al., 2019; Lao et al., 2019; Rodrigues et al., 2019; Shiohama et al., 2019); and evaluating cognitive functioning (Virji-Babul et al., 2013). All these cited researchers used non-invasive brain registration techniques and extended studies that had the same objectives but used traditional paradigms (Contestabile et al., 2010; Wiseman et al., 2015). Early studies that focused on examining, for example, the relationship between AD and people with DS, identified certain limitations. The most notable limitation is the variability of the degree of intellectual disability of the assessed person and the problems understanding the verbal instructions of the test among people with severe or profound intellectual disability (Crayton et al., 1998; Oliver, 1999). In addition, the behavioral differences associated with dementia have an impact not only on the person with DS but also on the professionals who administer the tests, so the reliability of these responses must be examined and psychometrically guaranteed (Oliver et al., 2011). Finally, it should be noted that many psychometric scales have not been validated in populations with AD and SD, but some studies have made progress in addressing these gaps (Dekker et al., 2015).

Thus, our proposal in the present work is to advance in the knowledge of all the present contributions of brain signal neuroimaging techniques in the DS population. Many studies have shown the advantages of using non-invasive brain registration techniques, as these measurements have no response bias or learning processes because when tasks are used, they have already been learned in the preregistration phases. The use of the main techniques such as EEG (electroencephalography), MRI (magnetic resonance imaging), PET (positron emission tomography), DTI (diffusion tensor imaging), and fMRI (functional magnetic resonance imaging) provide information on structural mechanisms and functional aspects, explaining the connectivity networks that can be found in healthy subjects as well as those suffering from diseases (Massoud and Gambhir, 2003; Hoehn and Aswendt, 2013; Aswendt et al., 2017).

Regarding the SD population, the systematic review presented by Neale et al. (2018) indicates that PET techniques allow the identification of amyloid accumulation prior to the onset of Alzheimer's disease (AD), while techniques based on EEG and MRI identify cognitive impairment and can be assessed as biomarkers for the detection and diagnosis of AD in this population.

In addition to the already cited signals, in the general population, fMRI registers have been used in the last 20 years as derivatives of the images obtained from MRI data, and certainly, the use of fMRI to assess brain activity and functioning, as well as the use of various study designs, has become common (Welvaert and Rosseel, 2014). Despite the amount of published works, we believe that it is urgent to advance this field since the data obtained from fMRI are highly valuable and their use provides unique and remarkable results in general and specific populations.

One of the most interesting advantages of studying the DS population with resting-state fMRI, as with other non-invasive techniques, is that the resting-state fMRI register does not depend on the intellectual level of the person being evaluated. In this approach, the person should only be at rest inside a resonator, without doing anything special, with his or her eyes open and without moving. In contrast, in other study designs, the fMRI signal is recorded when a specific cognitive task (e.g., language, memory, motor, among others) is being performed. Clearly, based on the uses and potential of the fMRI signal in resting-state designs (Lu et al., 2007; Biswal et al., 2010), this approach may be an interesting option because it allows the analysis of the cognitive activity of people with ID and, as in the general population, facilitates the systematic study of spontaneous fluctuations of the BOLD signal. Notably, information from studies that involve a task is derived from a direct source of activation when the participant is faced with the task set, and the resting-state data are derived from an indirect source that is not associated with the task.

It seems clear that some of the problems that occur when using fMRI signals may be aggravated for the DS population. This observation is based on the difficulties that people with DS have been reported to have when MRI data is being obtained, which suggests that with fMRI techniques, such the occurrence incidents can increase. Reviews of MRI in the DS population indicate several questions that can be applied to fMRI studies, which we can summarize in the following points. (1) It is feasible that the brain connectivity network in DS persons is more weakened than that in healthy persons of the same chronological age. We must interpret exactly what we mean by the weakening of a network, as the points of interest to determine it are diverse (e.g., density, laterality, entropy, and complexity, among other possible topics). It is also possible that the manifestation of cognitive impairment in persons with DS will, in some cases, be compensated by the intervention of other brain areas. (2) Brain volume and head size are smaller in DS persons than in healthy populations (Pinter et al., 2001; Rodrigues et al., 2019), and a smaller number of neurons and fewer synaptic extensions and altered neuronal differentiation in fetuses with DS are detected (Takashima et al., 1981; Bhattacharyya et al., 2009; Kanaumi et al., 2013). This issue has led to the use of DARTEL (Ashburner, 2007) as a template in some studies with persons with DS (Lin et al., 2014). DARTEL (Diffeomorphic Anatomical Registration through Exponentiated Lie Algebra) is a specific brain template used in the preprocessing phase or analysis of fMRI data to take into account deformations that must be parameterized by a single flow field, which is considered to be constant in time. (3) The DS population moves excessively during the registration of the signal, which leads to many experimental difficulties (Pujol et al., 2014). In this sense, Lao et al. (2019) point out an extremely important fact: the MRI signals acquired with motion correction below 1% allow the use of a general template for one of the general populations, which facilitates the study of PET signals, among others. If these assessments are documented by MRI data, it seems logical to consider them with respect to fMRI records.

Given the aforementioned points, it seems that the use of MRI data in the DS population is an interesting matter. Consequently, the question whether fMRI signals are adequate for use in this population must be addressed. The study of spontaneous fluctuations of the fMRI BOLD signal has become the goal of investigating connectivity and understanding how brain networks are organized, whether based on a stimulus response or simply at rest (Fox and Raichle, 2007; Raichle, 2009). Moreover, resting-state fMRI (rs-fMRI) has become an increasingly popular method of MRI that investigates synchronous activity across regions in the absence of an explicit signal correlation-based task (Corbetta, 2012; Snyder and Raichle, 2012). Data acquired by fMRI provides valuable information for explaining the determinants of network dysfunction, either with task designs to evaluate the person's different cognitive abilities (Hampson et al., 2006) or with rs-fMRI in populations that may have some diseases such as epilepsy (Centeno and Carmichael, 2014).

Therefore, based on the extensive contributions that have been shown in robust fMRI studies and the detailed review of non-invasive neuroimaging techniques in the population with DS (Baburamani et al., 2019), it is now important to establish the best experimental option to guarantee the validity and reliability of the brain signals recorded in individuals with DS. Moreover, our intention is to provide a reference point that allows us to systematically accumulate and order the available information and the findings derived from the fMRI and DS binomial (other signals such as PET or EEG are contemplated in the review of Neale et al., 2018, but this is not the case for fMRI signals.) Therefore, we want to review the works published to date in relation to fMRI data and people with DS, provide useful insights that identify the main difficulties and findings that researchers could utilize, and discuss the different ways to solve these difficulties.

## Method

### Search of Published Studies

**Figure 1:**
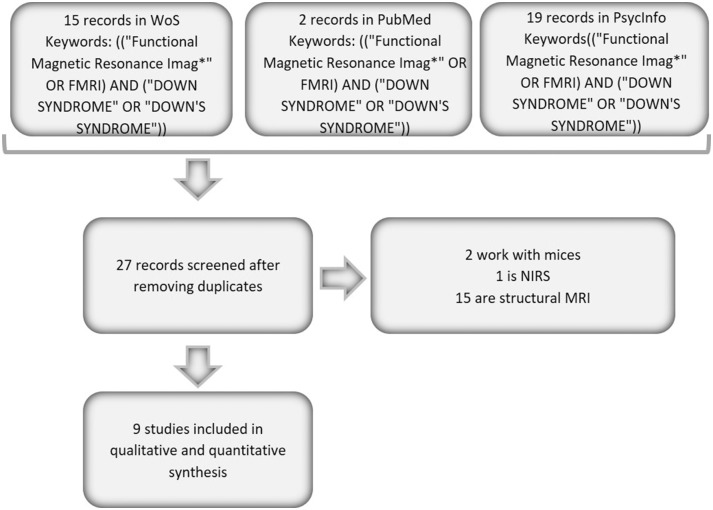
Flow chart of the analyzed papers.

The articles included in the present study were searched in the Web of Science (WoS), PubMed and PsycInfo databases. The following inclusion criteria were applied: the articles had to be original fMRI papers that included a sample of persons with DS and that were published from 1992 to October 17, 2019. The literature search was conducted using a Boolean algorithm with the following keywords: (“DOWN SYNDROME” OR “DOWN'S SYNDROME”) in the title and (“Functional Magnetic Resonance Imag^*^” OR FMRI) in any part of the paper. If we added the keyword Alzheimer, no works were found in the three databases; consequently, in the present study, we worked with a combination of DS and fMRI studies. The search was performed independently by three researchers, and we obtained a 100% rate of agreement between them for the search; all papers found by the three researchers were considered in the study. Following these search criteria, we identified a total of 15 papers in WoS, 2 papers in PubMed and 19 records in PsycInfo. After duplicates were removed, a total of 9 papers were screened. None of these papers were discarded; thus, 9 articles were fully reviewed and were included in the current study (identified with an ^*^ in the bibliography). Figure 1 presents a graph that summarizes this search process.

Three independent researchers analyzed each paper to estimate the most important information from each of the included articles. Table 1 shows the main characteristics of these articles.

**Table 1:** Main characteristics of the analyzed papers.

**Title**	**Authors**	**Year**	**Journal**
Abnormal brain synchrony in Down Syndrome.	Andesrson J. S., Nielsen J. A., Ferguson M. A., Burback M. C., Cox E. T., Dai L. et al.	2013	NeuroImage
Violence: heightened brain attentional network response is selectively muted in Down syndrome.	Anderson J. S., Treiman S. M., Ferguson M. A., Nielsen J. A., Edgin J. O., Dai L. et al.	2015	Journal of Neurodevelopmental Disorders
Functional magnetic resonance imaging of cognitive processing in young adults with Down syndrome.	Jacola L. M., Byars A. W., Chalfonte-Evans M., Schmithrst V. J., Hickey F., Patterson, B. et al.	2011	American Journal on Intellectual and Developmental Disabilities
Functional magnetic resonance imaging of story listening in adolescents and young adults with Down syndrome: evidence for atypical neurodevelopment.	Jacola L. M., Byars A. W., Hickey F., Vannest J., Holland S. K. and Schapiro M. B.	2014	Journal of Intellectual Disability Research
Abnormal fMRI activation pattern during story listening in individuals with Down syndrome.	Reynolds Losin E. A., Rivera S. M., O'Hare E. D., Sowell E. R. and Pinter J. D.	2009	American Journal on Intellectual and Developmental Disabilities
Functional magnetic resonance imaging shows aberrant language lateralization in Down syndrome.	Seyffert M., Field K. and Pinter J.	2002	Annals of Neurology
Resting-state functional connectivity in individuals with Down syndrome and Williams syndrome compared with typically developing controls.	Vega J. N., Hohman T. J., Pryweller J. R., Dykens E. M., and Thornton-Wells T. A.	2015	Brain Connectivity
The effectiveness of the computerized visual perceptual training program on individuals with Down syndrome: An fMRI study.	Wan Y.T., Chiang C. S., Chen S. C. and Wuang Y. P.	2017	Research in Developmental Disabilities
Differential effects of Down's syndrome and Alzheimer's neuropathology on default mode connectivity.	Wilson L. R., Vatansever D., Annus T., Williams G. B., Hong Y. T., Fryer T. D. et al.	2019	Human Brain Mapping

As shown in Table 1, the nine works were published after 2002, and seven studies were published in the last 9 years. Finally, the work of Seyffert et al. (2002) is a conference proceeding and therefore provides little information.

## Results

### Main Characteristics of the Studies

Table 2 shows the main characteristics of the samples used in the nine analyzed works. As shown in the table, seven of the works are American, one is Taiwanese, and one is from the United Kingdom. The total sample sizes range between 6 and 76 participants belonging to two groups, a DS group and a control group; however, in Jacola et al. (2014) and Vega et al. (2015), three groups are used. In Jacola et al. (2014), there are two control groups, one paired by chronological age and the other by mental age; in Vega et al. (2015), there is a group of DS individuals, a group of Williams syndrome (WS) participants and a control group. In general, no characteristics of the total sample are provided; however, sample characterization is performed for each of the groups. The percentage of males, in general, is higher than 50% in both analyzed groups, even reaching 71.05% in the study by Wan et al. (2017). Notably, in the eight studies in which information on the age of the participants is available, the ages range between 5 and 47 years old. In the majority of the papers, the groups were paired by chronological age and/or sex.

**Table 2:** Sample description of the analyzed papers.

**Paper**	**Sample Country**	***n***	**% men**	**Groups**	**n by group**	**% men by group**	**Age mean by group**	**Sampling**	**Matching**
Anderson et al. (2013)	USA	29	58.62	2	DS: 15 C: 14	DS: 60.00 C: 57.14	DS: 20.20 C: 23.70	Accidental	Chronological age and sex
Anderson et al. (2015)	USA	29	58.62	2	DS: 15 C: 14	DS: 60.00 C: 57.14	DS: 20.20 C: 23.70	Accidental	Chronological age and sex
Jacola et al. (2011)	USA	25	60.00	2	DS: 13 C: 12	DS: 61.54 C: 58.33	DS: 18.30 C: 19.00	Accidental	Chronological age and sex
Jacola et al. (2014)	USA	36	47.22	3	DS: 11 C_CA: 13 C_MA: 12	DS: 54.54 C_CA: 53.85 C_MA: 33.33	DS: 18.3 C_CA: 18.3 C_MA: 5.4	Accidental	C_CA: chronological age C_MA: mental age
Reynolds Losin et al. (2009)	USA	18	50.00	2	DS: 9 C:9	DS: 44.44 C: 55.56	DS: 16.5 C: 17.8	Accidental	Chronological age
Seyffert et al. (2002)	USA	6		2	DS: 3 C: 3			Accidental	
Vega et al. (2015)	USA	68	60.29	3	DS: 10 WS: 18 C: 40	DS: 40 WS: 72.22 C: 60.00	DS: 38.98 WS: 25.89 C: 46.95	Accidental	
Wan et al. (2017)	Taiwan	76	71.05	2	DS: 38 C: 38 The DS group was divided in two samples: intervention (*n* = 18) and control (*n* =20)	DS: 71.05C: 71.05	DS: 13.17 C: 13.07	Accidental	Chronological age and sex
Wilson et al. (2019)	United Kingdom	54	50.00	2	DS: 34 C:20	DS: 55.00 C: 47.00	DS: 43.5 C_CA: 43.5	Accidental	Chronological age

*DS, Down syndrome group; C, control group; C_CA, control group matched by chronological age; C_MA, control group matched by mental age; WS, Williams syndrome group*.

Notably, in the work of Wan et al. (2017), the DS group is divided into two groups, an intervention group (*n* = 18) and a non-intervention group (*n* = 20). Because of this subdivision, the groups are not equal in terms of sex and age, so the proportion of men in the DS intervention group is 61.11%, while in the DS non-intervention group, the proportion of men is 80%; the average age in the DS intervention group is 14.09 years, while in the DS non-intervention group, it is 12.35 years. Finally, none of the nine studies present information on the degree of disability of the persons with DS.

### fMRI Description and Main Results

As shown in Table 3, in eight of the works, the resonator used is 3 Teslas, while in the oldest work (Seyffert et al., 2002), the resonator is 1.5 Teslas. In five studies, the block design is used; therefore, the subjects must perform a task while undergoing BOLD signal acquisition. The rest of the works use the resting-state paradigm, but two of them employ unusual strategies, such as presenting visual stimuli during signal acquisition (Anderson et al., 2013, 2015). Jacola et al. (2011, 2014) and Reynolds Losin et al. (2009) use semantic listening type tasks, whereas in the Wan et al. (2017) study, visual perception tasks are used. The amount of time that persons are in the resonator performing the task is generally short, ranging from 5 min 30 s in the shortest case to 50 min in the longest case. In general, the objective of the analyzed works is to determine if there is a differentiated activation pattern between the DS group and the analyzed control group. Clearly, the papers estimating functional connectivity networks try to show the difference between groups in relation to network patterns and characteristics.

**Table 3:** Principal characteristics of the fMRI design and principal results obtained.

**ID**	**Teslas**	**Design**	**Task**	**Task time**	**Main aim**	**Principal results**
Anderson et al. (2013)	3T	Resting-state imaging with visualizing cartoons		50'	Compare fMRI scans of 15 individuals with Down syndrome to 14 typically developing control subjects while they viewed cartoon video clips	Measurements of subject motion were significantly higher in Down syndrome subjects than in controls. Down syndrome subjects showed higher levels of synchrony between distributed brain networks as well as between the vast majority of gray matter regions. Down syndrome subjects exhibited weaker correlations only for a relatively small subset of the most correlated regions, whether negatively or positively related. Regardless of the distance separating the regions, pairs of regions that showed anticorrelation in a large control sample showed increased correlation (reduced anticorrelation) in Down syndrome.
Anderson et al. (2015)	3T	Resting-state imaging with visualizing cartoons		50'	Examine functional brain activation in response to stylized violence stimuli in Down syndrome and in typically developing individuals to determine whether regional brain activation patterns could be characterized, as well as whether atypical neural activation might be present that could provide clues to a basis for the deficits seen in Down syndrome	In typically developing individuals, the brain's dorsal attention network was most active during violent scenes in the cartoons; this was significantly and specifically reduced in Down syndrome participants. Individuals with Down syndrome exhibited significantly reduced activation in the primary sensory cortices, and such perceptual impairments may constrain their ability to respond to more complex social cues such as violence.
Jacola et al. (2011)	3T	Block design	Paradigm that required participants to make a decision based on semantic information derived from visually presented stimuli	5'30”	Understand the relationship between cognitive processing and brain activation in individuals with Down syndrome on tasks that measured aspects of both verbal and visual-spatial abilities	A significant difference was present in task performance between the mean of DS and control individuals; the mean of DS individuals was inferior to that of controls. In relation to fMRI, controls had 13 areas activated, whereas DS had 20 areas activated.
Jacola et al. (2014)	3T	Block design	Language processing: a passive story listening paradigm	5'30”	Explore neural activation during language processing in participants with DS compared with typically developing groups matched for chronological and mental age	Random effects group analyses documented a reduced activation magnitude in the DS cohort than in both control groups. The pattern of activation within the DS cohort additionally included significantly greater activation in the midline frontal regions (BA 9 and 10) and cingulate gyri (BA 23, 24, 30 and 32).
Reynolds Losin et al. (2009)	3T	Block design	Passive story-listening task (Blocks: forward, backward and rest)	6'08”	Investigate whether individuals with DS exhibit aberrant language-related activation patterns compared to an approximately age-matched typically developing control group during an easily performed passive story-listening task	Control > DS: Forward > Backward—Right middle temporal gyrus. DS > Control: Forward > Rest—Right precuneus; Backward > Rest—Right precuneus.
Seyffert et al. (2002)	1.5T	Block design	Silent naming of pictures of common objects presented through fiber-optic goggles. As a control condition, subjects viewed pixilated images of the same objects with instructions to look without attempting to name them		Unspecific objective related to language deficits in Down syndrome	Greater activation was observed in the right inferior frontal and right superior temporal gyrus in the DS group than in the controls. Vega et al. (2015)
Vega et al. (2015)	3T	Resting state		5'	First aim: confirm previous findings of increased between-network connectivity in DS individuals compared with TD controls and determine whether such differences are specific to DS or are also observed in another developmental disability disorders, such as WS. Characterize how the within-network connectivity profiles of DS and WS could be compared with each other and with TD participants. Together, these aims are intended to support the replication of previous work while providing new insights into resting-state brain function across two different neurodevelopmental disorders.	The results showed that alterations of between-network connectivity, particularly in the DMN, are a characteristic of a number of neurodevelopmental disorders involving intellectual disability, including DS and WS. Perhaps within-network connectivity is a feature that shows more variable patterns across different neurodevelopmental disorders.
Wan et al. (2017)	3T	Block design	Two types of visual perceptual tasks: two-choice revised version of Hooper Visual Organization Test (T-HVOT) and Full Picture Matching Test (FPMT)	6'42”	(1) Develop and implement a one-year computerized visual perceptual training (CVPT) program for DS, (2) use a standardized visual perception assessment to evaluate the effectiveness of the CVPT program, and (3) examine the changes of cortical activation patterns of DS individuals after one-year of CVPT intervention by utilizing functional magnetic resonance imaging (fMRI)	The results showed that the DS intervention group had significant improvements in TVPS-3 after the intervention. The fMRI results indicated more activation in the superior and inferior parietal lobes (spatial manipulation), as well as the precentral gyrus and dorsal premotor cortex (motor imagery) in the DS intervention group. In the T-HVOT vs. FPMT comparison, TD individuals showed highly significant bilateral activations in the middle occipital gyrus, middle temporal gyrus, middle frontal gyrus, and inferior frontal gyrus.
Wilson et al. (2019)	3T	Resting state	Eyes closed while awake	10'	(a) Determine the potential functional connectivity alterations of the DMN in people with Down syndrome; (b) examine the relationship between DMN connectivity and age, IQ and performance on memory and executive function tasks in people with Down syndrome; and (c) investigate differences in DMN connectivity in people with Down syndrome with and without fibrillar Aβ accumulation, indicative of Alzheimer's disease neuropathology	The Down syndrome (all) group did not display a typical profile of DMN connectivity; almost no anti-correlation with other cortical regions was observed. Disrupted functional connectivity of the DMN is an early biomarker of Alzheimer's disease neuropathology.

A summary of the results found in the different works is also provided in Table 3. The task description of these works is noteworthy since none of them mention the previous learning periods, and the level of difficulty of the task is not indicated in all of them. In fact, in older works using a resting-state approach, visualization is used to avoid excessive movement. The extent to which these sequences would be considered resting is debatable, since these types of records, as we have reiterated, occur in the absence of any external stimulation. An examination of the concrete results of each of the papers indicates some common characteristics, even though the papers are not strictly comparable. In general, differential activation patterns are seen in the DS samples compared to the control groups. This pattern is not regular, and statistically significant differences are observed in unilateral comparisons in both the Down > Control and Control > Down assessments. Most likely, depending on the characteristics of the tasks, distinct and small extrapolated activation patterns were obtained. For example, Seyffert et al. (2002) found greater activation in the DS sample than in the control group in a task to silently name pictures of common objects. On the other hand, Reynolds Losin et al. (2009), in a passive story-listening task (Blocks: Forward and Backward and Rest), found similar effects in some comparisons. For example, in the Forward > Backward task, statistically significant effects were found in the unilateral comparisons of the Control > DS groups. However, in other tasks (Forward > Rest), the statistically significant difference was in the opposite direction DS > Control. The works of Jacola et al. (2011, 2014) were especially consistent since they only found statistically significant effects in the Control > DS comparisons in all the areas studied. Inconsistent and irregular activation patterns, as we mentioned, can be seen in the remaining works (Vega et al., 2015; Wan et al., 2017; Wilson et al., 2019). The two works carried out with a resting-state approach (Anderson et al., 2015) showed less activation in the functional connectivity networks presented in the DS samples. However, these results are not comparable since both studies used resting-state techniques with the presentation of visual stimuli.

## Discussion

We should wait a few more years for more studies using fMRI techniques to be published and to include various specific populations. Even so, it is quite pertinent to promote the use of these techniques, which deserve special attention in the DS population for their singularities in relation to their cognitive functioning and intellectual level. Generally, the increase in the use of neuroimaging techniques has led to the appearance of many underpowered studies with small sample sizes, which leads to many missed results (Button et al., 2013). This leads us to continue to explore the different findings.

Presently, it is optimistic to talk about generalized conclusions given the small amount of evidence available (eight or nine papers depending on the consideration of Seyffert et al., 2002). In any case, we aim to provide useful reference points to support future work that utilizes fMRI techniques with the DS population.

First, there is evidence of functional and structural differences between populations. Lower brain volume and lower activity (activation) recorded by fMRI appear to be typical in the DS population. It is evident that the morphological differences in the brain of a person with DS brain fuel the discussion about the normalized atlases, which were also revealed in the MRI studies of persons with DS. For instance, Pujol et al. (2018) used SPM voxel-based morphometry (VBM) with DARTEL algorithms in the image preprocessing phase.

Second, related to the last point, in fMRI sessions, we have already reiterated that the recording problems stem primarily from the movement of the person within the resonator. This problem is common in children and in populations with pathologies that compromise motor control (Aranyi et al., 2017). This tendency is observed among DS persons. As a consequence, some records have to be eliminated, or statistical routines are required to correct broadband movement in the preprocessing phase. It is a source of noise to consider in this type of work. Except for the work of Wilson et al. (2019), this issue is not mentioned in the rest of the works. In this regard, the proposals of Ciric et al. (2017) should be taken into account in all fMRI studies and with more intensity in high-movement populations such as persons with DS. Even if their suggestions do not provide specific corrections for DS samples, they have been shown to be effective in reducing the perverse effects of excess movement. The work of Wilson et al. (2019) may be a reference for the appropriate application of these corrections.

Third, in the same manner, the sample sizes are small. This finding is not new; it is a recurring theme in many works, not necessarily just DS studies. Therefore, this factor is not a distinctive aspect of these samples. The difficulties of sampling are well-known but are not different from many other proposals for fMRI. From a classical perspective, the expected minor sampling error in this work would not be inferior to 0.1124 assuming a CI of 95% and a theoretical parameter π = 0.5.

From a methodological point of view, and as the fourth issue, we can identify certain doubts in the configuration of control groups of healthy people in the employed designs. It is important to note that the mental development of subjects with intellectual disability is not the same as that of healthy subjects of equal chronological age but should not differ significantly when matched for their mental age (Carducci et al., 2013). The focus is on the selection of an appropriate control group, and the options are matching by chronological age or by mental age. In the first case, we consider a non-specific maturation process, and in the second, we address cognitive skills, which are also developmental but focus fundamentally on performance. Notably, in the case of studies that involve tasks, it would be appropriate to promote having control groups matched by mental age, as it is a question of comparing cognitive performance and brain signals (Jacola et al., 2014). Only one study analyzed described two paired control groups, one by chronological age and another by mental age, because basal functioning was being evaluated rather than factors associated with an explicit cognitive task or component. In this respect, there is one aspect that is of little or no consideration. If a control group is generated from the estimation of mental age matching, strict matching must be performed (consisting with having an exact mirror in the control group of each case in the experimental group). If a poorly matched group is used due to sampling difficulties, the control is usually verified by comparing the means of the mental age between the two groups; relevantly, IQ ranges differ greatly between individuals with and without DS. In people without ID, the IQ ranges between 85 and 115 in almost 66% of people (according to the normal curve properties); the population of ID individuals is usually distributed into those that exhibit slight or medium delays, that is, those with IQs up to 70 in most cases. In addition, IQs are stable in the adult population but much less stable in the child population; thus, comparison with this type of matching can be misleading (Amador and Forns, 2019). No matching analyses have been reported with empirical evidence of homoscedasticity between groups paired with lax criteria.

In this sense, as a fifth point, the works analyzed here show comparisons between groups of statistically significant activations or networks obtained from the fMRI records during a semantic recognition task, passive listening, non-verbal denomination of objects, a visual perception task or a resting state (see Table 3). In all cases, activation differences are reported in certain areas of the brain with intergroup comparison. The fact that the visual areas are activated less in the DS sample than in the control group or that there are differences in other areas due to visual stimuli does not indicate specific properties of the DS population. The same consideration must be addressed in network differences. In fact, in general, all studies agree in reporting a lower activation intensity in DS groups than control groups, and the study by Wan et al. (2017) presents an interesting intragroup comparison that does describe intrinsic properties of the DS population. However, the lower activation level obtained in the DS group than in the controls confirmed the effects of brain impoverishment described above. We can make many comparisons with many different stimuli; however, these comparisons will not directly lead us to discovering unique brain behavior properties of the DS population. In fact, an activation increment is described in some connections, and a decrement is noted in others in the comparisons between networks in DS samples and the paired control groups. However, these differences do not allow the establishment of a stable and regular pattern typical of DS people.

These findings lead us to another crucial point. The use of tasks that previously published papers have used are tasks adapted to the peculiarities of the target population. Comparison with a healthy control group of similar chronological age does not in any way support characteristic outcomes among the DS population. The comparison between DS and non-DS groups does not provide relevant evidence regarding individuals with DS. It seems more methodologically reasonable to make intragroup comparisons than intergroup comparisons and, when feasible, use internal classification criteria for the identification of subgroups. For instance, to compare older and younger individuals, those with higher cognitive competence and those with lower competence, those with a higher cognitive reserve level and those with a lower level and so on within the DS population, any other criteria that allows characterization of cognitive competence by signals and study of its distribution should be utilized.

Intragroup comparisons present other types of statistical issues that extend beyond the objective of this paper but are reasonably resolved and can be used in DS studies. We can observe examples of these issues in AD or Parkinson's populations or in populations with other pathologies with characteristic intellectual deficits, such as Williams syndrome, Fragile FXS syndrome, Rett syndrome and Turner's syndrome (Beaton et al., 2010; Thornton-Wells et al., 2010; Chai et al., 2012; Stevenson et al., 2012; Venuti et al., 2012; Klabunde et al., 2015; Reynolds et al., 2015).

While previously mentioned, it is surprising that there are scarce resting-state fMRI records. Several studies have shown the relationship between the default mode network (DMN) and healthy aging processes; thus, the estimation of this type of well-known network should also be a good vehicle for the study of brain functioning through fMRI in the DS population (Farràs-Permanyer et al., 2019), which leads to a final point that we must propose. The study of brain signals in pathologies with cognitive deficits is common; however, it is not commonly used to evaluate people with DS, and the lack of studies with this approach cannot be explained by the difficulties of sampling or the workload of registration alone. The sampling difficulties and workload are the same those for other pathologies, but there are more published studies on people with other diagnosis than on people with DS.

The issue is that if fMRI (or other types of signals) data are used as indicators of cognitive status, they are likely to be perceived as being unnecessary in these (and other) cases. However, this is not the essential question. The conception of the brain signal cannot currently be conceived as the study of a specific activity associated with a stimulus. The nature of brain function requires a much broader consideration than designs associated with tasks in which the statistical detection of increased activation in a specific area of the brain through intergroup or intragroup comparisons is a priority. Our proposal is based on the need to study functional and effective connectivity networks for the whole brain at rest and in series that are not less than 5 min, according to the recommendations of Cole et al. (2010) and Van Dijk et al. (2010). This recommendation provides a measure of brain functionality (i.e., connectivity networks) in a short and feasible time and with no bias.

## Conclusions

To conclude, we want to summarize the notable points discuss above. First, it is feasible to use fMRI signals in a population with DS, as long as measures are provided with the utmost rigor. Second, it seems preferable to use the resting-state paradigm in this population. Third, it would be beneficial to invest time and effort in studying how the brain signal in the population with DS could be used as a biomarker of cognitive activities. Fourth, this type of data must be exhaustively analyzed to estimate classification and discrimination functions between differential groups within the same population of DS. Techniques such as linear or non-linear discriminant analysis and latent profile analysis, among others, may be especially relevant. Finally, we must remember that the techniques related to movement reduction can help improve recruitment and sampling, especially reducing experimental mortality due to register errors. All these questions are very limited thus far with respect to the effort to meta-analyze data from fMRI signal studies in populations with DS.

## Data Availability Statement

The datasets generated for this study are available on request to the corresponding author.

## Author Contributions

All authors have contributed to all parts of the paper: in the selection of works, the extraction of information and analysis and the writing and review of the work.

## Conflict of Interest

The authors declare that the research was conducted in the absence of any commercial or financial relationships that could be construed as a potential conflict of interest.
